# Synthetic DNA and biosecurity: Nuances of predicting pathogenicity and the impetus for novel computational approaches for screening oligonucleotides

**DOI:** 10.1371/journal.ppat.1008649

**Published:** 2020-08-06

**Authors:** R. A. Leo Elworth, Christian Diaz, Jing Yang, Paul de Figueiredo, Krista Ternus, Todd Treangen

**Affiliations:** 1 Department of Computer Science, Rice University, Houston, Texas, United States of America; 2 Department of Microbial Pathogenesis and Immunology, Texas A&M Health Science Center, Bryan, Texas, United States of America; 3 Department of Veterinary Pathobiology, Texas A&M University, College Station, Texas, United States of America; 4 Norman Borlaug Center, Texas A&M University, College Station, Texas, United States of America; 5 Signature Science, LLC, Austin, Texas, United States of America; University of Wisconsin Madison, UNITED STATES

## Overview

DNA synthesis technologies are enabling rapid advancements in the field of synthetic biology, which involves the design and fabrication of novel biological components. The immense promise of DNA synthesis technology is unmistakable, but so is its potential for intentional or accidental misuse. In the interest of biosecurity, the United States Department of Health and Human Services (HHS) issued its Screening Framework Guidance for Providers of Synthetic Double-Stranded DNA in 2010, which calls on commercial providers of double-stranded DNA (dsDNA) to voluntarily screen all orders. Most notably, a group of dsDNA synthesis companies known as the International Gene Synthesis Consortium (IGSC) has implemented the Harmonized Screening Protocol (HSP) in alignment with the HHS Guidance. While there is not a single DNA screening algorithm used by all IGSC members, DNA-screening software typically follows HSP guidance by aligning a query sequence to a relatively short list of biological toxins and select agent genomes, genes, or proteins. We herein describe challenges involved in the current screening process, ideas for improvements, and examples that illustrate why current obstacles to advancement are so critical to overcome.

## Current DNA screening guidelines

The HHS Guidance [1] and the HSP [2] have set a precedent for corporate responsibility and oversight within the DNA synthesis industry, but the HHS Guidance also recognizes its own inherent technical limitations [1]. At a high level, the HSP flags sequences as threats if they show high similarity to unique proteins, genes, or genomic regions within the US Select Agents and Toxins List, the European Union Control List of Dual-Use Items, the Australia Group List, and (as applicable to US foreign distribution) agents on the Export Administration Regulations Commerce Control List (CCL). Conversely, sequences that do not show high homology are cleared for synthesis and distribution to approved customers. The main advantages of this approach are its simplicity and alignment with existing federal regulations. The clear disadvantage, as recognized within the HHS Guidance itself, is the exclusion of biosecurity threats outside of the pathogens and toxins that fall under the Select Agent Regulations (SAR), which is currently administered by HHS/Centers for Disease Control and Prevention (CDC) and the US Department of Agriculture (USDA)/Animal and Plant Health Inspection Service (APHIS). While HHS commends ongoing development of better screening practices, it justifies this broad exclusion from current guidance because the determination of pathogenicity is a complex and ongoing area of research.

## Nuances of predicting pathogenicity

**Fig 1:**
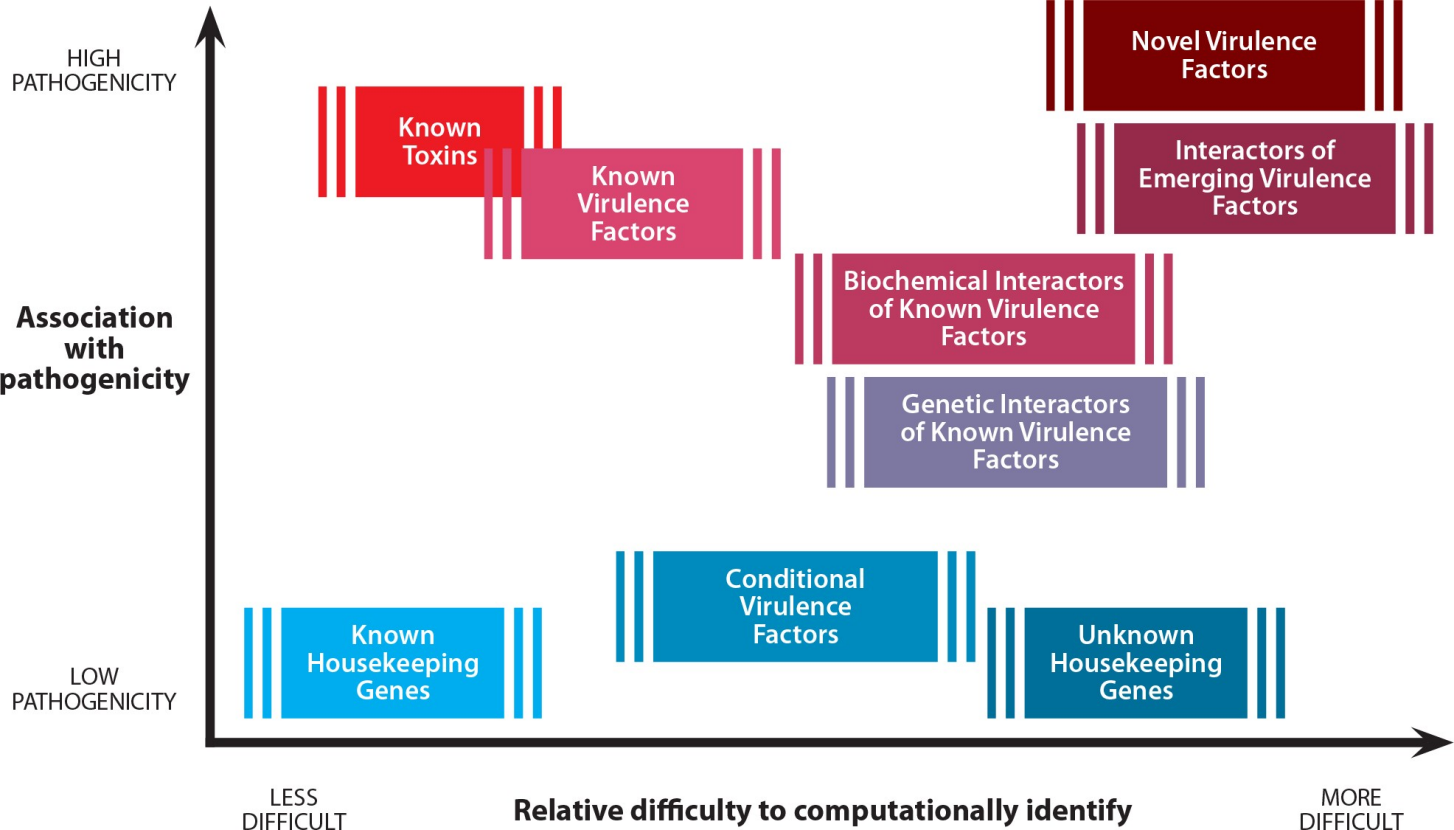
DNA screening challenges. The *y* axis shows the known or potential pathogenicity of a gene or virulence factor, while the *x* axis displays the corresponding difficulty of identifying the pathogenicity level through automated computational tools. The combination of pathogenicity and the ease of its computational inference is a complex interplay between how much we already know about established virulence factors and how well we can characterize newly emerging ones, as well as their specific functions.

The HHS Guidance notes that pathogenicity is a complex and ongoing area of research with no regulatory precedent, which makes it challenging to predict and integrate into sequence screening guidelines [https://www.ncbi.nlm.nih.gov/books/NBK50869/] (Fig 1). However, significant strides have been made toward understanding the virulence mechanisms underlying microbial pathogenesis. Rather than wait for pathogenesis to be fully understood by the research community before incorporating functional information into DNA-screening software and guidelines, near-term goals should be identified to begin integrating this information in a step-wise manner as research advances. The following four vignettes highlight the importance of looking beyond taxonomy when assessing pathogenicity: (1) housekeeping genes in pathogens, (2) pathogenic sequences in typically harmless organisms, (3) virulence factors in host-specific pathogens, and (4) genes only dangerous in concert.

### Housekeeping genes in pathogens

As mentioned in the HHS Guidance, although pathogens must contain virulence factors to cause disease, many genes within pathogens are harmless. Such examples include “housekeeping genes,” which are required for viability and maintenance of basal cellular functions [3]. There is a wealth of resources available for the identification of known housekeeping genes, including schemes developed for “gold-standard” multilocus sequence typing (MLST). These schemes are stored in large, accessible databases such as PubMLST [4] and use known alleles of housekeeping genes to identify a wide range of bacteria (e.g., pathogens, commensals, or industrial use). While known housekeeping genes are easier to detect than those currently unknown, scientific progress is unnecessarily impeded if any housekeeping genes are falsely identified as pathogenic and thus as potential biosecurity concerns.

### Pathogenic sequences in typically harmless organisms

Taxonomic, functional, and environmental information is valuable to interpret indirect contributions to pathogenesis, such as the relative pathogenicity of proteins in opportunistic pathogens or newly emerged virulence functions in commensals. Opportunistic pathogens like *Candida albicans* are usually harmless, but they can cause disease in hosts with weakened immune systems [5]. The hyphal form of *Candida albicans* is more invasive than its yeast form and key to its pathogenicity. A number of genes, including the agglutinin-like sequence protein Als3, are up-regulated during candidiasis infection and hypha formation [6], demonstrating that environmental cues can accentuate the virulence potential of a commensal. Beyond opportunistic pathogens, experimental evidence has shown that new virulence functions can evolve in commensals [7]. This can naturally occur through the acquisition of new accessory virulence genes on plasmids, such as the horizontal gene transfer of botulinum neurotoxin-encoding plasmids from *C*. *botulinum* to nonpathogenic species *C*. *sporogenes* and *C*. *butyricum* [8], or by mutating existing genes to increase virulence. Synthetic biology has broadened the range of these mutational possibilities.

### Virulence factors in host-specific pathogens

Host ranges and targets are critical to consider when assessing biosecurity concerns because a subclass of pathogens are specialists, or agents that can parasitize only a small number of hosts. This parasitic property boils down to the molecular mechanisms underlying an agent’s pathogenicity and specificity to certain hosts. One example is the *Plasmodium* genus, which consists of unicellular eukaryotes responsible for the development of malaria. Its host specificity can partially be understood by comparing different versions of the circumsporozoite protein (CSP) among multiple *Plasmodium* species that infect different hosts. CSP is responsible for the binding and invasion of liver cells by sporozoites. The CSP of the human parasite *P*. *falciparium* efficiently binds to the human hepatoma cell line (HepG2 cells); however, the CSPs of parasite species targeting other hosts, such as rodent-targeting *P*. *yoelii* (pyCSP) or simian-targeting *P*. *knowlesi* (pkCSP), more deficiently bind to HepG2 cells by factors of 17,800 and 4,790, respectively [9]. Therefore, a DNA screening protocol focused on human risks would justifiably not consider the detection of pyCSP or pkCSP sequences to be concerning.

### Genes only dangerous in concert

When a sequence is a component of a larger pathogenic complex, another dimension is added to the DNA screening process. Some virulence factors do not pose a biosecurity threat on their own and must work together with other factors to create a pathogenic function (e.g., by forming protein complexes). For example, hemolysin BL (HBL) is a three-component complex found among the strains of *Bacillus cereus*, a pathogen responsible for some foodborne illnesses. HBL is considered the primary virulence factor in *B*. *cereus*, with hemolytic, dermonecrotic, and vascular permeability activities[10]. Its constituent proteins, the lytic components L1 & L2 and the binding component B, are encoded by the genes *HblC*, *HblD*, and *HblA*, respectively [10]. This complex is formed when the three proteins bind to the host cell membrane in an ordered, sequential manner [11], and it is incapable of materialization if any of the three proteins are absent. Fully understanding the interactions and complexes of proteins resulting in pathogenicity is a long-term research goal, but in the near term, known complexes could be incorporated into screening software to lay the groundwork for expanding such information with new research findings.

## Computational challenges and path forward

Despite the availability of functional information in public databases, major technical gaps are preventing this information from being fully utilized in DNA screening software (Fig 1). While there is no single query tool that can precisely and efficiently connect a short (<200 bp) [12], unknown full or partial gene sequence with its most likely protein match, Gene Ontology (GO) terms, and other functional annotation information from disparate public databases, including more recent tools like double index alignment of next-generation sequencing data (DIAMOND) [13] and InterProScan [14] offer promising building blocks for future approaches. Taken together with taxonomic predictions and what is known of its protein targets, such functional predictions can provide immediate benefit to pathogenicity assessments. More long-term goals include predicting how multiple proteins behave together to form a pathogenic function, how mutations impact virulence functions, the effect of specific environments or host immune system variations on pathogenicity, the influence of host range on threat assessments, and how machine learning can help predict unknown functions in poorly annotated sequences.

Our four vignettes are far from comprehensive, but they serve to show that pathogenicity is a complex phenomenon that cannot merely be captured through the use of static taxonomic lists. We hope to foster dialogue around the nuances in this area that could contribute to the development of future tools to improve biosecurity best practices. Rather than introduce additional bureaucratic complexity to scientific research, we hope more sophisticated computational approaches will enable scientists to streamline their ability to align with evolving biosecurity policies. It will be a community effort to move our biosecurity guidelines forward [12], but the rapid advances in synthetic biology and genome editing require our willingness to make incremental steps beyond the classic model of taxonomic biothreat classification and regulation.
